# Lectin Recognition Patterns in the Gut of *Meccus (Triatoma) pallidipennis* and Their Association with *Trypanosoma cruzi* Metacyclogenesis

**DOI:** 10.3390/microorganisms13081823

**Published:** 2025-08-05

**Authors:** Berenice González-Rete, Juan Antonio López-Aviña, Olivia Alicia Reynoso-Ducoing, Margarita Cabrera-Bravo, Martha Irene Bucio-Torres, Mauro Omar Vences-Blanco, Elia Torres-Gutiérrez, Paz María Silvia Salazar-Schettino

**Affiliations:** Departamento de Microbiología y Parasitología, Facultad de Medicina, Universidad Nacional Autónoma de México, México City 04510, Mexico; bere.gonzalez.ciencias@gmail.com (B.G.-R.); anton_avina@ciencias.unam.mx (J.A.L.-A.); oard_2000@yahoo.com.mx (O.A.R.-D.); imay@unam.mx (M.C.-B.); marbu@unam.mx (M.I.B.-T.); movences@gmail.com (M.O.V.-B.)

**Keywords:** metacyclogenesis, *Trypanosoma cruzi*, Chagas disease, triatomine midgut, triatomine proctodeum, parasite-vector interaction, glycoproteins, proteomic maps, lectin-blots

## Abstract

The successful transmission of Trypanosoma cruzi, the causative agent of Chagas disease, depends on intricate interactions with its insect vector. In Mexico, *Meccus pallidipennis* is a relevant triatomine species involved in the parasite’s life cycle. In the gut of these insects, the parasite moves from the anterior midgut (AMG) to the posterior midgut (PMG), where it multiplies. Finally, *T. cruzi* differentiates into its infective form by metacyclogenesis in the proctodeum or rectum (RE). This study aimed to characterize and compare the protein and glycoprotein profiles of the anterior midgut (AMG) and rectum (RE) of *M. pallidipennis*, and to assess their potential association with *T. cruzi* metacyclogenesis, with special attention to sex-specific differences. Insects were infected with the *T. cruzi* isolate ITRI/MX/12/MOR (Morelos). Protein profiles were analyzed by polyacrylamide gel electrophoresis, while glycoproteins were detected using ConA, WGA, and PNA lectins. The metacyclogenesis index was calculated for male and female triatomines. A lower overlap of protein fractions was found in the RE compared to the AMG between sexes, suggesting functional sexual dimorphism. Infected females showed greater diversity in glycoprotein patterns in the RE, potentially related to higher blood intake and parasite burden. The metacyclogenesis index was significantly higher in females than in males. These findings highlight sex-dependent differences in gut protein and glycoprotein profiles in *M. pallidipennis*, which may influence the efficiency of *T. cruzi* development within the vector. Further proteomic studies are needed to identify the molecular components involved and clarify their roles in parasite differentiation and suggest new targets for disrupting parasite transmission within the vector.

## 1. Introduction

Chagas disease is a zoonotic disease endemic to 22 countries in the Americas, from the southern United States to Argentina and Chile, affecting 6–8 million people and causing approximately 50,000 deaths annually [1,2,3]. The disease, caused by the protozoan parasite *Trypanosoma cruzi*, develops in a complex ecological environment involving intra- and interspecific interactions between vector, parasite, and host [4]. While vector-borne transmission accounts for over 90% of infections in endemic regions [5], other less important modes include blood transfusion and congenital transmission [6,7].

Thirty-four species of triatomine have been identified in Mexico [8,9,10,11]. *Meccus pallidipennis* is one of the main vectors of *T. cruzi*, not only because of its ability to adapt to different climatic conditions—which allows it to be widely distributed in thirteen states of the country—but also because of its entomological parameters, which influence its efficiency in transmitting the parasite [10,12,13]. Triatomines have a highly specialized digestive system divided into the anterior midgut (AMG), posterior midgut (PMG), and proctodeum or rectum (RE). The morphological and biochemical characteristics of this anatomical region facilitate their hematophagous mode of life and allow various interactions with the parasite throughout its cycle within the vector [14,15,16]. After ingesting infected blood, *T. cruzi* passes through these intestinal regions where it undergoes key processes, such as differentiation into epimastigotes, multiplication, and metacyclogenesis [17,18,19].

After blood ingestion, erythrocyte lysis occurs in the AMG of the vector and approximately 80% of the parasites die [20,21]. Those that survive move on to the AMG, where they anchor to the perimicrovillar membrane (PMM), which facilitates their multiplication by binary fission, and finally reach the RE, where metacyclogenesis takes place. Metacyclogenesis is an essential process for parasite transmission, as it transforms the epimastigote into the metacyclic trypomastigote, which is the infective form in the mammalian host [22,23].

This process varies with vector species and parasite strain [24,25]. It has been suggested that this interaction may be regulated and/or mediated by biochemical factors, such as interactions between glycoconjugates on the surface of the parasite and the cells of the vector’s gut [25,26,27]. In addition to facilitating these interactions, glycoconjugates play key roles in digestion, defense, and parasite differentiation [28,29,30].

Previous studies have demonstrated the presence of glycoproteins in the triatomine gut. ConA, PNA, and WGA are lectins commonly used to detect these molecules due to their broad affinity for certain carbohydrate residues that include glucose (α-D-glucose) and mannose (α-D-mannose), N-acetylgalactosamine-galactose (D-GalNAc-Gal), N-acetylglucosamine (D-GlcNAc), and sialic acid (Neu5Ac) [31,32]. These glycoconjugates are involved in critical physiological processes and may influence the establishment and development of *T. cruzi* in the vector [33,34]. However, the relationship between protein glycosylation patterns in the gut and metacyclogenesis has not been extensively studied. The RE, where parasite differentiation occurs, is particularly relevant for investigating the molecular interactions that support this process.

In this study, we aimed to characterize and compare the protein and glycoprotein profiles of two key gut regions—the anterior midgut (AMG) and the rectum (RE)—in *Meccus pallidipennis*, with a particular focus on sex-specific differences. These regions were analyzed in both infected and uninfected insects to explore potential associations with *T. cruzi* metacyclogenesis. By examining the expression patterns of proteins and glycoconjugates and analyzing differences between sexes, we aimed to provide insights into the gut environment that may influence parasite differentiation and transmission. This approach may contribute to a better understanding of the molecular context in which parasite development occurs, offering a foundation for future studies aimed at interrupting vector–parasite interactions.

## 2. Materials and Methods

### 2.1. Meccus Pallidipennis Specimens

Thirty male and thirty female *M. pallidipennis* specimens from a colony derived from triatomines collected in the state of Morelos, Mexico, were used. The adults were divided into two groups: control and infected; each consisted of 15 females and 15 males. Both groups were kept under controlled conditions at 28 °C, 60% relative humidity, and a 12/12 h light/dark cycle in the insectarium of the Laboratory of Parasites Biology, Department of Microbiology and Parasitology, Faculty of Medicine, Universidad Nacional Autónoma de México.

### 2.2. Infection of Adult M. pallidipennis Specimens

The insects in the control group were fasted for 15 days, while those in the experimental group were infected by feeding on female CD-1 mice (15–18 g) previously inoculated with 20,000 blood trypomastigotes of the ITRI/MX/12/MOR (Morelos) *T. cruzi* isolate [35]. The triatomines were infected 15 days after mouse inoculation, coinciding with the exponential growth phase of *T. cruzi* [36]. To confirm blood ingestion, triatomines were individually weighed before and after feeding. They were then identified and kept under controlled conditions [37]. The animal study protocol was approved by the Institutional Ethics Committee of the Faculty of Medicine, UNAM (protocol code: FM/DI/078/2020, approved on 4 August 2020). Both control and experimental groups were maintained under identical experimental conditions.

### 2.3. Trypanosoma Cruzi Isolate

Isolate ITRI/MX/12/MOR, named “Morelos”, was first obtained from a male *M. pallidipennis* trapped in Cuernavaca, Morelos, in 2012 and characterized as TcI [38]. This isolate was maintained in CD-1 mice through programmed cyclic passages.

### 2.4. Confirmation of Infection

Fifteen days after feeding on infected mice, the rectal contents of each triatomine were collected by compression and examined microscopically to confirm the presence of *T. cruzi* in the feces [39]. Ten microliters of PBS pH 7.2 and 5 μL of triatomine feces were placed on a microscope slide. The sample was homogenized, and 10 μL was taken for observation under a microscope with a 40X objective (Olympus CH-2, Center Valley, PA, USA) [36].

### 2.5. Dissection and Extraction of Meccus pallidipennis Gut

Triatomines from the control and infected groups were anesthetized on an ice bed, dissected, and processed for extraction of AMG and RE 15 days after infection [35]. The limbs of each specimen were removed with dissecting tweezers. The abdomen was disinfected with 70% alcohol. The connective tissue was identified and cut to expose the peritoneal cavity, and the Malpighian tubules and adipose tissue were removed. Both regions were then washed five times with 1 mL of sterile PBS and protease inhibitors (cOmplete^TM^, Mini Cocktail, Roche, Mannheim, Germany; PMSF, Thermo Scientific^TM^, Waltham, MA, USA) and stored at −80 °C with 100 μL of solution A with protease inhibitors (KCl 19 mM, MgCl_2_ 1.1 mM, NaH_2_PO_4_ 0.7 mM, Na_2_HPO_4_ 0.2 mM, Complete Mini 4X; pH 7.4) until processing [32].

### 2.6. Extraction, Precipitation, and Quantification of AMG and RE Proteins

Tissue samples were thawed; grouped by experimental group, sex, and intestinal region; and subjected to disruption using a method described elsewhere, with minor modifications [40]. Briefly, samples were subjected to three cycles of sonication at 20% amplitude for 20 s using a sonicator (Branson Ultrasonics^TM^ Sonifier^TM^ SFX150 Cell Disruptor, St. Louis, MO, USA), interspersed with cooling periods at 4 °C for 30 s.

Each analyzed sample corresponded to a pool of 15 *M. pallidipennis* specimens. The protein extract from each group was precipitated with a solution of 10% trichloroacetic acid (TCA) and 20 mM dithiothreitol (DTT) in cold acetone (Deatherage Kaiser, 2015) [41]. Proteins were resuspended in 100 μL of solution A containing protease inhibitors. Total protein was quantified by the bicinchoninic acid (BCA) colorimetric method [42] using the Pierce^®^ BCA Protein Assay Kit (Thermo Scientific, Cat. No. 23225) according to the manufacturer’s instructions.

### 2.7. Protein Profile by SDS-PAGE Electrophoresis (1D)

Protein profiles in the AMG and RE of adult *M. pallidipennis* specimens were determined by sodium dodecyl sulfate-polyacrylamide gel electrophoresis (SDS-PAGE) [43]. Precast gels with a 4–12% concentration gradient (NuPAGE^TM^ 4–12% Bis-Tris Gel, 1.0 mm × 12 wells, Invitrogen, Ref. NP0322BOX, Carlsbad, CA, USA) were used, loading 20 μg of protein per well. Broad molecular weight markers (10–250 kDa) were used as reference (Precision Plus Protein^TM^ Dual Color Standards, Bio-Rad; Cat. 1610374, Hercules, CA, USA). Electrophoretic migration was performed in an electrophoresis chamber (Mini Gel Tank, Invitrogen^TM^ Thermo Fisher Scientific) at 200 V for 45 min. Upon completion, the gel was stained with Coomassie Blue (R-350).

### 2.8. Proteomic Mapping by Isoelectric Focusing and Two-Dimensional (2D) Electrophoresis

For isoelectric focusing, 7 cm immobilized pH gradient (IPG) strips with a linear 3–10 pH interval were used (IPG Strips, Bio-Rad, Cat. 1632000). Each strip was loaded with 80 μg protein from male or female, AMG or RE, using a rehydration solution consisting of 7 M urea, 2 M thiourea, 4% CHAPS, 60 mM DTT, 2% IPG buffer (Cytiva; Cat. 17600087, Marlborough, MA, USA), and 0.2% bromophenol blue [32]. Isoelectric focusing was performed in a PROTEAN i12 IEF cell system (Bio-Rad) in three steps: (1) 250 V for 15 min, (2) 4000 V for 60 min, and (3) 4000 V until 15,000 V/h was reached. The strips were then loaded onto preformed 4–12% concentration gradient polyacrylamide gels (NuPAGE^TM^ 4–12% Bis-Tris ZOOM^TM^ Gel, 1.0 mm × IPG well, Invitrogen; Cat. NP0330BOX) and electrophoresed in a chamber at 200 V for 45 min. Gels were stained with colloidal Coomassie Blue to visualize proteins [44].

### 2.9. Glycosylation Patterns from Lectin Blots

Following 1D or 2D electrophoresis, proteins were transferred onto PVDF membranes (Immobilon^®^-P, Merck Millipore; Cat. IPVH00010, Burlington, MA, USA) using a Transfer-Blot Turbo^TM^ system (Bio-Rad) at 15 V for 30 min. Membranes were blocked with 0.1% BSA in PBS-T (0.03% Tween-20) for 1 h at room temperature and then incubated with peroxidase-conjugated lectins diluted in the same blocking buffer: ConA (0.5 μg/μL, *Canavalia ensiformis*, Cat. L6397), WGA (0.5 μg/μL, *Triticum vulgaris*, Cat. L3892), and PNA (1 μg/μL, *Arachis hypogaea*, Cat. L7759) (all from Sigma-Aldrich, St. Louis, MO, USA). Membranes were washed sequentially with 0.1% Triton X-100 in PBS-T, PBS-T alone, and PBS (pH 7.2), with each wash performed seven times. Lectin binding was visualized using 2 mM 3,3′-diaminobenzidine (DAB) and 0.01% H_2_O_2_.

### 2.10. Gel and Blot Analysis

We imaged 1D and 2D gels and PVDF membranes on a Gel Doc^TM^ XR Imaging System (Bio-Rad Molecular Imager^®^). Molecular weight determination of proteins in 1D gels and blots and band analysis were performed using ImageLab v.6.1 (Bio-Rad). The generated master patterns were analyzed using PDQuest v.7.0.4 (Bio-Rad).

### 2.11. Parasite Count

The rectal contents of the triatomines were homogenized in 100 μL sterile PBS (pH 7.2), and the total number of parasites was estimated by counting in a Neubauer chamber in duplicate [35].

### 2.12. Determination of Metacyclogenic Index

Rectal samples were smeared in duplicate. Ten microliters of sample were smeared onto a microscope slide and allowed to dry at room temperature. The smears were fixed in methanol (CH3OH ≥ 99.8%, J.T. Baker^TM^) and stained using the Giemsa technique (Sigma-Aldrich GS500). The slides were examined under a 40X objective (Olympus CH-2, Center Valley, PA, USA) and 100 parasite forms, including epimastigotes and metacyclic trypomastigotes, were counted. In cases where 100 parasite forms were not found, the total number of parasite forms counted was considered as 100%. Metacyclogenic index was defined as the fraction of metacyclic trypomastigotes present and expressed as a percentage [45].

### 2.13. Statistical Analysis

The Kolmogorov–Smirnov (K-S) test and the Levene test were used to assess normality and homoscedasticity, respectively. The Mann–Whitney U test was used to determine differences between the number of total parasites found in males and females and the number of metacyclic trypomastigotes based on the metacyclogenic index. All analyses were performed using GraphPad Prism v.9.4.1. All data are expressed as the means of the total number of parasites and the number of metacyclic trypomastigotes ± standard deviation.

## 3. Results

### 3.1. Protein Profiles by SDS-PAGE Electrophoresis (1D) in AMG and RE of Male and Female Triatomines Infected with T. cruzi

**Figure 1:**
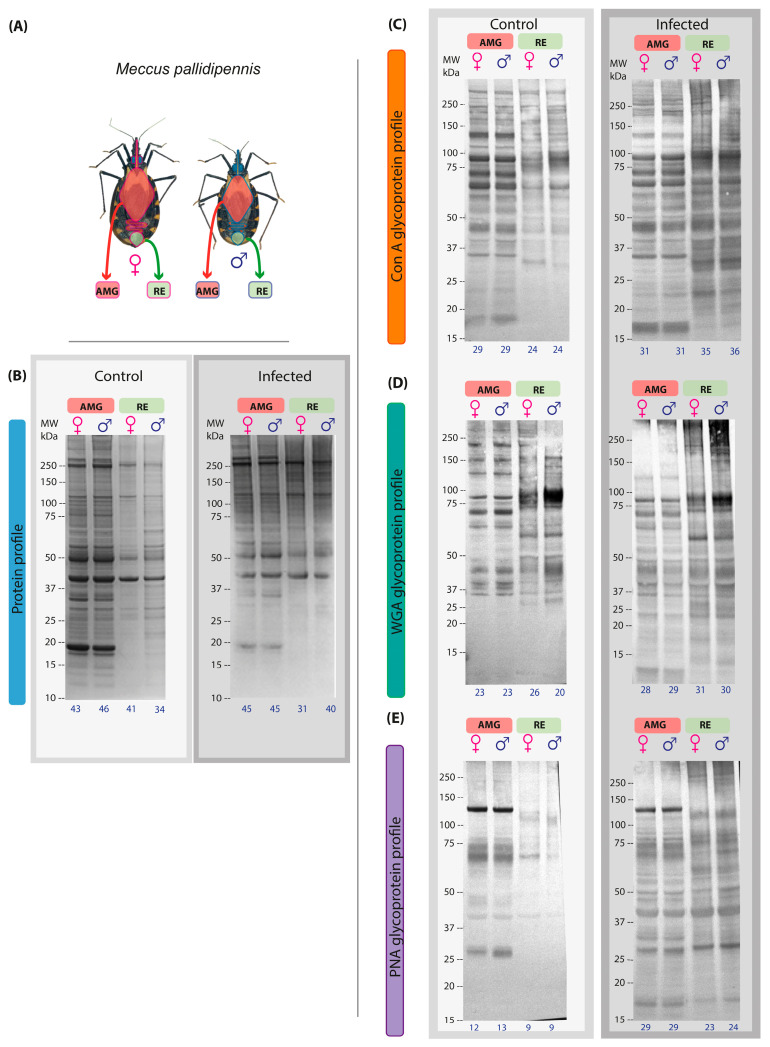
Protein and glycosylation profiles of AMG and RE samples from female and male *M. pallidipennis* uninfected and infected with *T. cruzi.* (**A**) AMG and RE of female and male M. pallidipennis. (**B**) AMG and RE protein profiles by SDS-PAGE. (**C**) AMG and RE glycosylation profile by ConA. (**D**) AMG and RE glycosylation profile by WGA. (**E**) AMG and RE glycosylation profile by PNA. MW = molecular weight; kDa = kilodaltons; AMG: anterior midgut; RE: proctodeum. The total number of bands identified in each experimental group is given at the bottom of each profile.

The AMG and RE samples of triatomines differed significantly in size (Figure 1A). The protein profile of the AMG and RE samples of triatomines infected with *T. cruzi* showed differences between the control and infected groups. The electrophoretic profile of AMG samples from females and males in the control group showed 51 protein components (12–250 kDa), while in the infected group, only 45 bands were observed in both sexes (Figure 1B). In the AMG protein profile of infected specimens, no bands of molecular weight less than 15 kDa were detected, and a reduced intensity of some bands, such as those of 18 and 35 kDa, was observed (Figure 1B). On the other hand, 46 protein components (11–250 kDa, Figure 1B) were detected in the RE samples of control males and females. In the RE samples of the infected group, both sexes showed a lower number of components, with 31 bands in females and 40 bands in males (25–250 kDa, Figure 1B). The higher-intensity bands observed in both sexes and groups corresponded to 40, 50, 114, and 248 kDa.

Regarding glycosylation profiles detected by lectins, glycosylated bands were identified in the AMG and RE samples of both infected sexes. The affinity to ConA allowed the identification of 31 bands in the AMG samples of both sexes, while 35 bands were observed in the RE samples of females and 36 in those of males; in both regions, the molecular weight of the bands ranged from 27 to 250 kDa. In the RE samples, a 146 kDa band was identified in males, while the presence of a 23 kDa band was more prominent in females; in contrast, the 34 kDa band showed greater intensity in males than in females (Figure 1C).

Glycosylation analysis by affinity with WGA identified 28 bands in the AMG samples of females and 29 in those of males, with molecular weights ranging from 10 kDa to 250 kDa. In the RE samples, 31 bands were detected in females and 30 in males, with molecular weights ranging from 11 kDa to 250 kDa. In the RE samples, two bands of 32 and 54 kDa were identified in females, while one band of 122 kDa was detected in males. In addition, a band at 90 kDa was observed with greater intensity in males than in females, while the band at 60 kDa was more intense in females than in males (Figure 1D).

Finally, affinity with PNA identified 29 bands in the AMG samples of females and males, with molecular weights ranging from 14 kDa to 250 kDa. In the RE samples, 23 bands were identified in females and 24 in males, with molecular weights ranging from 11 to 250 kDa. In the RE samples, only three bands at 171, 213, and 250 kDa were detected in males, whereas two bands at 21 and 33 kDa were detected in females. In addition, bands at 50 and 70 kDa showed higher intensity in males compared to females (Figure 1E).

### 3.2. Proteomic Maps of AMG and RE of T. cruzi-Infected Male and Female Triatomines

**Figure 2:**
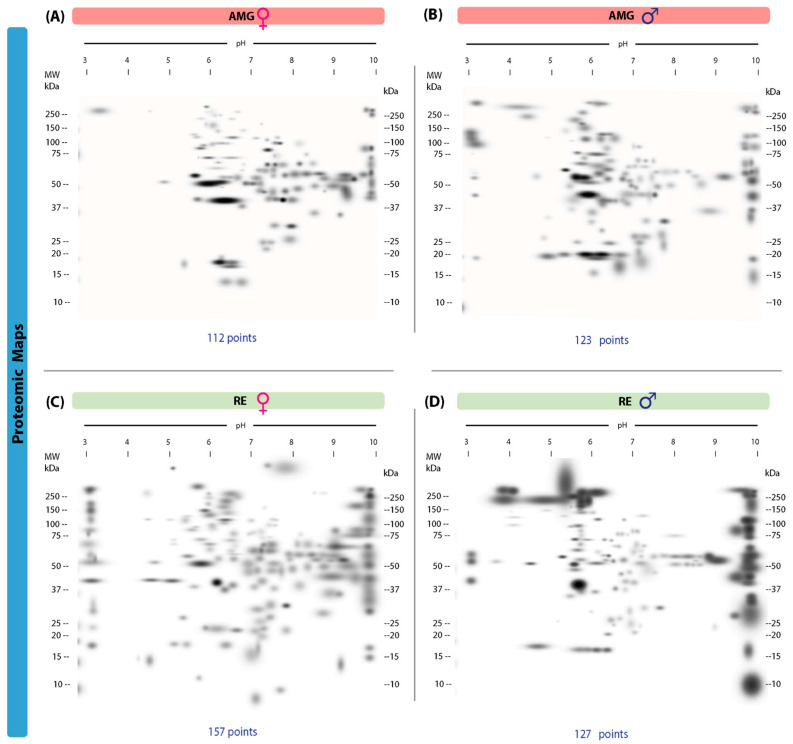
Proteomic master maps of the AMG and RE samples of female and male *M. pallidipenn* is specimens infected with *T. cruzi*. (**A**) AMG samples of females; (**B**) AMG samples of males; (**C**) RE samples of females; (**D**) RE samples of males. MW = molecular weight. kDa = kilodaltons; IP: isoelectric point. The total number of points detected is shown at the bottom of each map.

Proteomic maps of AMG and RE samples showed quantitative and qualitative (intensity) differences between the sexes in the infected group. In the AMG samples, infected females presented 112 protein spots, with 58 unique spots (13–250 kDa, IP of 3.14–9.99), whereas males showed 123 spots, with 69 unique spots (16–250 kDa, IP of 3.02–9.96) (Figure 2A,B). In contrast, 157 spots with 126 unique spots (10–250 kDa, IP of 3.05–9.96) were identified in the RE samples of females, while 127 spots, with 96 unique spots (10–250 kDa, IP of 3.05–10), were observed in males (Figure 2C,D). Fifty-four common protein spots were detected in the AMG samples and 31 in the RE samples from infected triatomines of both sexes.

### 3.3. Protein Glycosylation with α-Mannose and α-Glucose in the AMG and RE of T. cruzi-Infected Male and Female Triatomines

**Figure 3:**
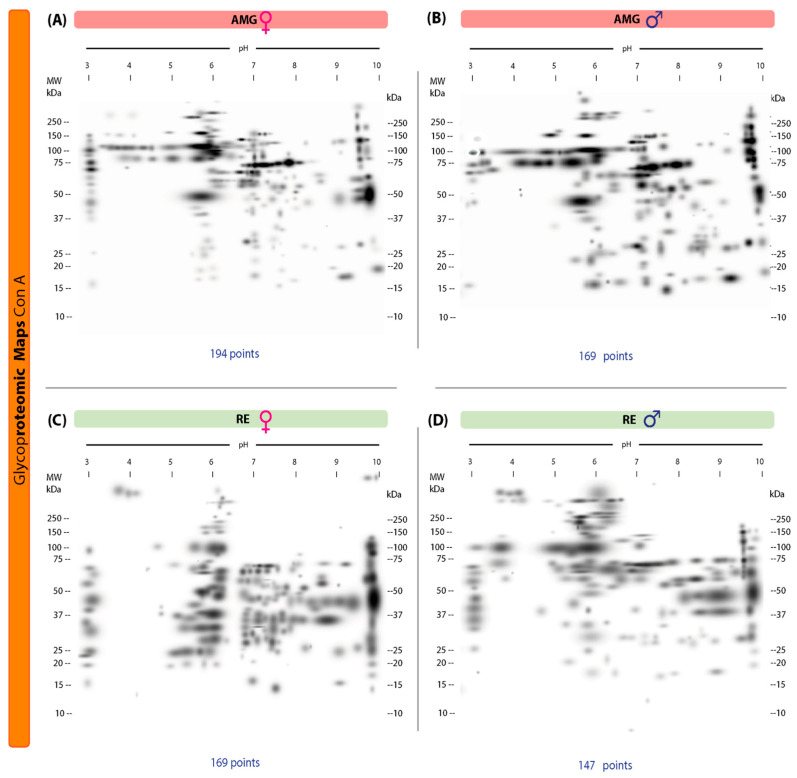
Proteomic master maps of the AMG and RE samples of female and male *M. pallidipennis* specimens infected with *T. cruzi* by affinity to ConA. (**A**) AMG samples of females; (**B**) AMG samples of males; (**C**) RE samples of females; (**D**) RE samples of males. MW = molecular weight; kDa = kilodaltons; IP: isoelectric point. The total number of points detected is shown at the bottom of each map.

Our 2D lectin blots revealed marked differences in the protein maps between infected females and males in both the AMG and RE samples. In the AMG samples of female triatomines, 194 protein spots with 103 unique spots and molecular weights of 16–250 kDa and IP values of 3.02–9.96 were detected (Figure 3A). In infected males, 169 spots and 78 unique spots with molecular weights of 15–250 kDa and IP values of 3.00–9.95 were found (Figure 3B). Of these spots, 91 were shared between the sexes. In RE samples, infected females showed 169 spots and 126 unique spots, with molecular weights of 15–250 kDa and IP values of 3–9.95, whereas in males, 147 spots and 104 unique spots with molecular weights of 10–250 kDa and IP values of 3.14–9.91 were detected (Figure 3C,D). Forty-three spots were shared by both sexes.

### 3.4. Protein Glycosylation with N-acetylglucosamine and Sialic Acid in the AMG and RE of T. cruzi-Infected Male and Female Triatomines

**Figure 4:**
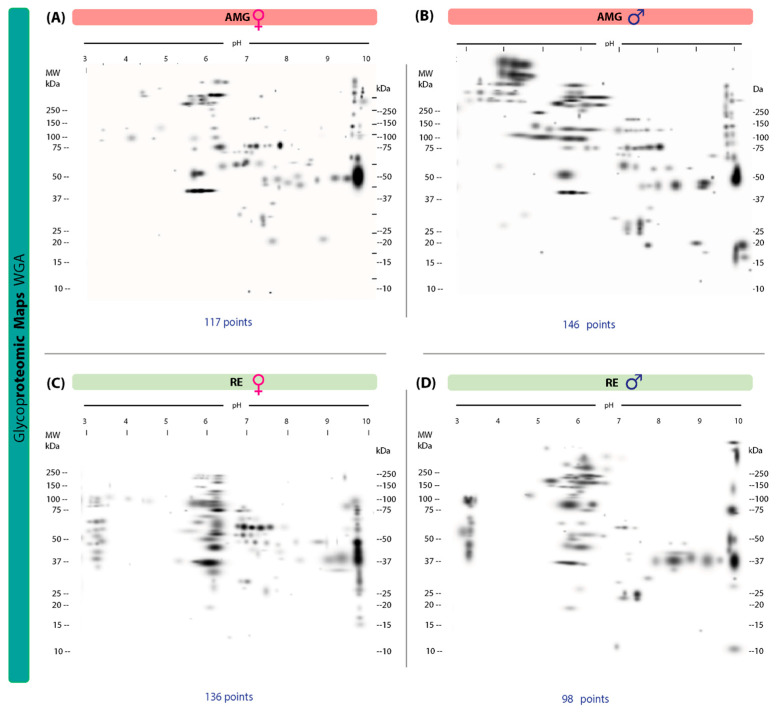
Proteomic master maps of the AMG and RE samples of female and male *M. pallidipennis* specimens infected with *T. cruzi*, by affinity to WGA. (**A**) AMG samples of females; (**B**) AMG samples of males; (**C**) RE samples of females; (**D**) RE samples of males. MW = molecular weight; kDa = kilodaltons; IP: isoelectric point. The total number of points detected is shown at the bottom of each map.

A total of 117 protein spots were identified in the 2D lectin blot revealed by WGA; 81 unique spots were identified in the AMG samples of infected females with weights of 10–250 kDa and IP values of 3.01–9.97 (Figure 4A). In infected males, 146 protein spots and 110 unique spots were detected, with a similar range of weights (10–250 kDa) and IP values of 3.02–9.98 (Figure 4B). Of these spots, 36 were shared by both sexes. In RE samples, females showed 136 spots with 104 unique spots (11–245 kDa, IP of 3–9.97) (Figure 4C); infected males showed 98 protein spots with 66 unique spots (10–250 kDa, IP of 3–10) (Figure 4D), with 32 spots shared by both sexes.

### 3.5. Protein Glycosylation with N-acetylgalactosamine and β-galactose in the AMG and RE of T. cruzi-Infected Male and Female Triatomines

**Figure 5:**
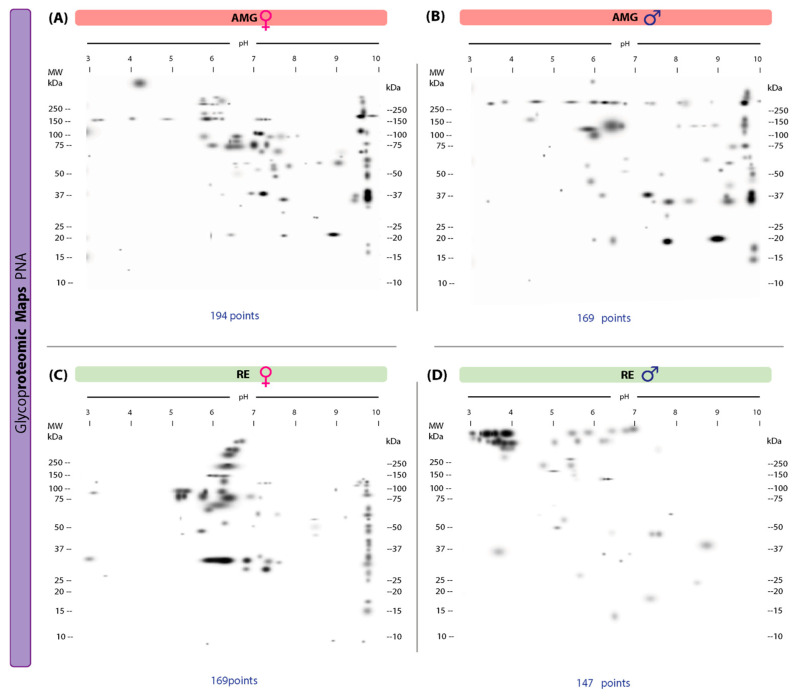
Proteomic master maps of the AMG and RE samples of female and male *M. pallidipennis* specimens infected with *T. cruzi*, by affinity to PNA. (**A**) AMG samples of females; (**B**) AMG samples of males; (**C**) RE samples of females; (**D**) RE samples of males. MW = molecular weight; kDa = kilodaltons; IP: isoelectric point. The total number of points detected is shown at the bottom of each map.

Ninety-one protein spots and 71 unique spots, with molecular weights ranging from 11–250 kDa and IP values of 3.00–9.99, were identified in the 2D lectin blots revealed by PNA of AMG samples from infected females (Figure 5A). In infected males, 72 protein spots and 52 unique spots with molecular weights in the same range (11–250 kDa) and IP values of 3.00–9.97 were detected (Figure 5B). Of these, 20 spots were shared by both sexes. In RE samples, females showed 74 spots with 70 unique spots (10–250 kDa, IP of 3–10) (Figure 5C); infected males showed 51 spots with 47 unique spots (14–250 kDa, IP of 3–9.84) (Figure 5D). Four spots shared by both sexes were detected.

### 3.6. Feeding, Parasite Burden, and Metacyclogenesis in Infected Triatomines

**Figure 6:**
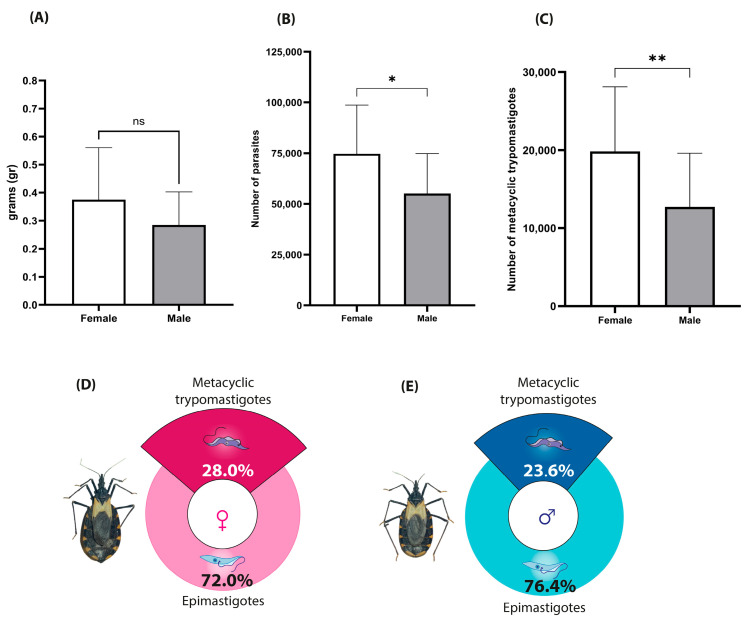
Feeding volume, parasite load, and metacyclogenesis index in infected *M. pallidipennis*. (**A**) Mean blood intake per insect, measured immediately after feeding; ± SD. (**B**) Total parasite counts in the rectal ampulla of individual insects at 15 days post-infection with *T. cruzi* (Morelos); Mean ± SD (* Mann–Whitney U test, *p* < 0.05). (**C**) Total number of metacyclic trypomastigotes estimated per insect; Mean ± SD (** Mann–Whitney U test, *p* < 0.05). (**D**) Metacyclogenesis index in infected females. (**E**) Metacyclogenesis index in infected males.

The analysis of feeding behavior showed that female *M. pallidipennis* ingested an average of 0.3750 ± 0.04 g of blood, whereas males consumed 0.2848 ± 0.03 g (U = 71, *p* = 0.2273; Figure 6A), with no statistically significant differences between sexes.

However, the parasite burden in the rectal ampulla differed significantly. Infected females harbored an average of 74,732 *T. cruzi* parasites compared to 55,044 in males (U = 52.5, *p* = 0.0360; Figure 6B), indicating a greater capacity for parasite proliferation in females.

Similarly, the metacyclogenic index was significantly higher in females (28%) than in males (23.6%) (U = 52, *p* = 0.0350; Figure 6D,E). This translated to an estimated 19,841 metacyclic trypomastigotes in females and 12,725 in males (Figure 6C), supporting a sex-associated difference in the efficiency of metacyclogenesis within the vector.

## 4. Discussion

In this work, the protein and glycoprotein profiles of the AMG and RE samples of male and female *M. pallidipennis* infected with *T. cruzi* were identified and described based on electrophoretic and lectin-binding patterns. Although mass spectrometry was performed, reliable protein identification was not achieved due to the absence of a species-specific genomic or proteomic database. The electrophoretic profile in the control group showed a wide range of molecular weights in both regions and sexes, suggesting a high protein diversity, possibly reflecting different physiological and structural functions [32,46]. In contrast, infected insects showed profiles with a lower number of bands, suggesting an infection-induced change in protein expression. These results are consistent with previous reports in the AMG [47]. This suggests that the presence of the parasite could shape the differential expression of proteins in the triatomine gut at different stages of its development and life cycle.

Our analysis showed a higher number of bands in the AMG samples of triatomines than in the RE samples, suggesting a higher proteomic diversity in this region; this could be related to the diversity of processes occurring during digestion, such as nutrient uptake, hormonal regulation, and other physiological processes relevant to the development of *M. pallidipennis* and related triatomines [21,48,49]. On the other hand, a reduction in the number of protein components was observed in the RE samples of infected insects, especially proteins of lower molecular weight (<25 kDa), suggesting that infection could degrade or inhibit the synthesis of certain proteins [50]. This alteration could be due to a strategy of the parasite to evade the immune system and favor its survival and differentiation within the vector [37,48,51,52,53]. Despite these differences, both regions of both experimental groups shared proteins with molecular weights of 40, 50, 114, and 248 kDa, indicating the presence of essential and/or constitutive molecules of the vector.

Post-translational modifications play a crucial role in the physiology of the insect and its interaction with the parasite [54,55,56,57]. In this context, the patterns of glycoprotein recognition by the lectins ConA, WGA, and PNA showed differences in terms of total protein profile, even among the same lectins. In particular, in the RE samples of both sexes, five differential bands were detected in males (ConA: 146 kDa; WGA: 122 kDa; PNA: 171 and 213; and 250 kDa) and four in females (WGA: 32 and 54 kDa; and PNA: 21 and 33 kDa), suggesting a possible role in parasite establishment in this region and highlighting the specificity of each lectin. The recognition patterns observed with ConA suggest the presence of complex glycan motifs rich in mannose/glucose residues, which are commonly associated with glycoproteins involved in immune modulation, cell adhesion, and modulation of the immune response, facilitating intestinal colonization of the vector and favoring metacyclogenesis [17,32,55].

Lectin binding observed with WGA, which has affinity for N-acetyl-D-glucosamine and sialylated structures, may indicate the presence of glycoproteins implicated in host–parasite interactions [17,32,55]. Although the ability of triatomines to synthesize sialic acid has been questioned, our results, in agreement with other studies, suggest that they do produce sialic acid [58,59,60,61]. Furthermore, in *T. cruzi* infection, only four common spots detected with PNA were observed in the RE samples, suggesting that the presence of the parasite alters the glycosylation pattern in this intestinal region [17]. These modifications could have implications for the stability and function of these molecules, favoring the interaction between parasite and transmitter. In other words, the individual glycosylation sites detected in the RE samples of both sexes could play a key role in the adhesion and colonization of *T. cruzi*, since the glycoproteins recognized by PNA could facilitate their attachment and establishment in the vector.

Lectin analysis also revealed significant differences between the sexes. Although both share glycoproteins required for AMG and RE function, changes in abundance and the occurrence of different components suggest specific adaptive responses to infection in both regions [31,32]. These changes could reflect sex-specific adaptive responses induced by the presence of the parasite and its possible role in insect adaptation, as well as specific biological functions [62]. In particular, the detection with PNA of unique components in the RE samples of females and males suggests that certain glycoproteins may play specific roles in sex-specific biology or in modulating the infection. However, given the observed differences between males and females in both infected and control groups, it is important to acknowledge that some of these differences may also arise from intrinsic physiological and anatomical distinctions between the sexes, independent of infection status. Therefore, interpretations regarding sex-specific infection responses must be made cautiously.

Previous studies have shown that the affinity of the lectins ConA, WGA, and PNA varies depending on the gut region and feeding conditions in *M. pallidipennis*. In fed insects, these lectins are most strongly recognized on the apical surface, basement membrane and cytoplasm of hindgut cells [61]. Similarly, in unfed fifth instar nymphs, a differential glycosylation profile was observed in the AMG and RE samples, related to key metabolic and structural functions [32]. Thus, different feeding conditions, and in our case infection, are modulating factors and determinants of glycoprotein expression in different regions of the triatomine gut.

Evaluation of the proteomic map of the AMG and RE samples of female and male triatomines infected with *T. cruzi* revealed significant variability in the expression of proteins and glycoproteins, including isoforms of the same protein, highlighting the complexity of the biological and physiological responses induced by infection and their possible association with metacyclogenesis (differential gene expression in epimastigotes leading to the formation of metacyclic trypomastigotes). The proteomic map of the AMG samples of infected females showed a total of 112 spots, while that of infected males showed 123 spots. A study focused on the proteomic map of the AMG samples of *M. pallidipennis* found that *T. cruzi* infection resulted in a greater number of spots and intensity of their expression compared to uninfected insects [47]. On the other hand, in unfed fifth-instar nymphs, 82 points were identified in the AMG samples and 98 in the RE samples, which are significantly lower numbers than our results. However, it should be noted that this stage is smaller than adults and that, as in the study by Nava-Mirafuentes et al. [47], the individuals analyzed were not infected [32]. Unlike previous studies that focused on unfed nymphal stages or uninfected individuals, our approach provides new insight into how *T. cruzi* infection, in combination with adult sexual dimorphism, modulates the glycoprotein composition in distinct gut regions. To our knowledge, this is the first comparative analysis integrating sex, infection status, and region-specific glycoprotein profiling in *M. pallidipennis*. It is important to note that although mass spectrometry analyses were conducted to identify specific proteins, these efforts were limited by the lack of a reference database for *M. pallidipennis*. Currently, our group is actively working on genome sequencing and de novo data generation to address this limitation in future studies.

In contrast, the proteomic map of the RE samples in infected females showed 159 points, while that of infected males showed 127. This variability, attributed to the presence of the parasite, could also be influenced by sex-dependent physiological factors. These results suggest that the glycoproteins in the RE seem to play a key role in the differentiation of *T. cruzi*, since it expresses a greater number of proteins than the AMG, which could explain the differences in susceptibility to the parasite and in the efficiency of metacyclogenesis between males and females. Both sexes showed 54 and 31 common points in the AMG and RE samples, respectively, which could represent a basic set of proteins involved in essential functions of these regions, regardless of sex. For example, some of these common points in the RE may correspond to the activity of locustatachykinin I (LomTK I) and locustatachykinin II (LomTK II); both neuropeptides play a role in muscle contraction in the proctodeum of *Rhodnius prolixus* during the processing of digested blood and excretion of products [63], including infectious forms. The presence of allatostatin might also be expected, which, unlike locustatachykinin, inhibits in vitro muscle contraction in the intestine of *R. prolixus*, including the rectum [64].

Our lectin analysis indicates that females express a greater number and diversity of proteins and glycoproteins in the AMG and RE, suggesting a differential response to the parasite. However, these differences may also reflect inherent physiological variations between sexes. The marked sexual dimorphism observed in the RE points to a specialized role of this region in metacyclogenesis, potentially involving proteins that modulate susceptibility and facilitate the efficiency of the process. In this context, the elevated protein expression observed in infected females may contribute to a higher rate of metacyclogenesis compared to males, supporting biological and physiological adaptations that enhance *T. cruzi* development and transformation. To strengthen the functional validity of these findings and complement the proteomic analysis, we propose that future studies include competition assays and glycosidase treatments to confirm the specificity of the observed interactions.

An example of this is the involvement of mucins such as Gp35/50 kDa in the specific adhesion of epimastigotes to the internal cuticle of the RE in triatomines such as *T. infestans* and *R. prolixus*. In vivo studies have shown that overexpression of these mucins increases infectivity and the number of metacyclic forms in the rectal ampulla [65]. This suggests that the higher protein expression observed in females may be related to the presence of molecules that promote parasite differentiation, thereby influencing the efficiency of the *T. cruzi* life cycle within the vector. It is possible that some of the different glycoproteins present in females increase the effectiveness of adhesion of Gp35/50 kDa or other mucins that play key roles in metacyclogenesis, facilitating parasite survival and transformation.

In this context, a crucial process takes place in the RE of triatomines: metacyclogenesis. This transformation is essential for the parasite, as it gives it the ability to become infective [22,66,67,68]. This morphogenetic transformation, in turn, triggers the release of different proteolytic and glycolytic enzymes in the RE of the insect, as well as the synthesis or degradation of proteins and their possible post-translational modifications [50,65].

Considering the above, this study determined the rate of metacyclogenesis in male and female *M. pallidipennis* specimens infected with the Morelos isolate of *T. cruzi*. The rate of metacyclogenesis was higher in infected females (28%) than in males (23.6%). This result is in agreement with previous reports, where females usually show higher rates [10,69]. However, in adults of *M. pallidipennis*, even with statistically significant differences, the percentage of metacyclic forms in feces is low compared to adults of other species, such as *T. dimidiata* or *T. barberi*; in *T. dimidiata*, an index of 38% was found for females and 16% for males, while adults of *T. barberi* had an index of 76.5%. This means that in terms of vectorial capacity, *M. pallidipennis* is a “poor vector” [70]. However, it is able to transmit the parasite effectively under different environmental conditions [13,71].

Salazar et al. (2005) [10] experimentally found a metacyclogenic index of 28% in *M. pallidipennis*, similar to that found in females in this study. Whether the index was determined in females or males was not specified in that paper, only that all insects were adults. Under natural conditions, that study reported an index of 15% in adults, slightly more than 50% of the metacyclic forms found in our experimental group. The above can be explained based on food availability and access, since food sources for triatomines in the wild are very diverse (including mammals and birds) to the extent that the establishment of the parasite may be different from that resulting from feeding in the laboratory [72,73,74]. Feeding frequency should also be considered. In the natural environment, triatomines can go days or even months without feeding, which reduces the population of *T. cruzi* in the rectal ampulla of infected insects [22,75,76].

In any case, the fact that females harbor a greater number of parasites has been attributed to the amount of blood they consume, which is greater than that ingested by males. This may be due to the needs associated with reproduction and egg production [71,73,76], although intrinsic anatomical and metabolic sex differences likely contribute as well. 

Finally, this study has certain limitations. While electrophoretic and lectin-binding analyses provided insights into glycoprotein profiles, the identity of specific proteins could not be determined due to the current lack of a species-specific database, despite efforts using mass spectrometry. Metacyclogenic index differences were observed between sexes, and associations with specific proteins remained. Our team is presently working on genome sequencing and de novo annotation to overcome this barrier. Moreover, while infection-related differences were highlighted, future studies should aim to more precisely discriminate infection-driven effects from those rooted in baseline sex-related physiology. In addition, the identities of differentially expressed glycoproteins remain unknown, and their role in *T. cruzi* adhesion or metacyclogenesis remain hypothetical at this stage.

Despite the crucial role of proteins and glycoproteins in the establishment of parasitic infections in insect vectors, our knowledge of the composition of these molecules and other components in triatomines is still very limited. Although mass spectrometry was performed in this study, the identification of specific proteins was hindered by the lack of an annotated reference genome or proteome for *M. pallidipennis*. Future efforts will focus on genome sequencing and de novo protein annotation to enable more accurate identification and functional characterization of the proteins involved in metacyclogenesis. This will allow the identification and quantitative characterization of proteins in terms of their structure, post-translational modifications, and interactions, with the aim of identifying glycoproteins potentially involved in metacyclogenesis, pending further functional validation in order to implement more effective control tools and/or strategies at the vector level.

## 5. Conclusions

Parasite numbers are higher in female *M. pallidipennis* specimens than in males, likely due to their greater blood intake and reproductive demands, which in turn reflect sex-specific biological adaptations and influence parasite development. *T. cruzi* infection is associated with changes in the expression patterns of proteins and glycoproteins, particularly in the RE, where infected females showed greater diversity, as revealed by electrophoretic and lectin-binding profiles. This differential expression may favor parasite survival, adhesion, and differentiation, ultimately contributing to a higher rate of metacyclogenesis in females.

The use of lectins (ConA, WGA, and PNA) revealed differential glycoprotein-binding patterns, highlighting the importance of post-translational modifications in the adaptation and response of the vector to infection. Each lectin detected a different number of glycoproteins, as well as diversity in their expression, demonstrating the complexity of these responses. The specific glycoproteins detected by PNA may play a key role in vector defense or adaptation to infection. In addition, the identification of shared protein and glycoprotein sites by both sexes suggests the existence of a core set of essential molecules that are conserved regardless of infection.

Although the specific identities of the differential proteins and glycoproteins remain to be determined, further detailed analyses may yield crucial information about their role in metacyclogenesis, with potential implications as targets in vector control strategies. The development of a reference genome for *M. pallidipennis* and subsequent mass spectrometry-based identification will be essential to confirm these findings and to enable the discovery of relevant biomarkers. However, further proteomic and glycoproteomic studies, including comprehensive characterization of post-translational modifications, are essential to fully understand the interaction between *T. cruzi* and its vectors, as well as its impact on metacyclogenesis.

## Data Availability

The original contributions presented in this study are included in the article. Further inquiries can be directed to the corresponding authors.

## Abbreviations

The following abbreviations are used in this manuscript:

AMG	Anterior Midgut
RE	Proctodeum or Rectum
ConA	Concanavalin A from Canavalia ensiformis
WGA	Wheat germ agglutinin from Triticum vulgaris
PNA	Peanut agglutinin from Arachis hypogaea

## References

[1] World Health Organization Chagas Disease (American Trypanosomiasis). https://www.who.int/health-topics/chagas-disease.

[2] Lidani K.C.F., Andrade F.A., Bavia L., Damasceno F.S., Beltrame M.H., Messias-Reason I.J., Sandri T.L. (2019). Chagas Disease: From Discovery to a Worldwide Health Problem. Front. Public Health.

[3] Hill J., Teal E., Cross C.L., Sanchez Z., Webber M.M., Oxborough R.M., Messenger L.A. (2024). Using iNaturalist Presence Data to Produce Suitability Maps for Triatoma Protracta, T. Rubida and T. Recurva in the American Southwest, Texas and Northern Mexico, to Identify Potential Transmission Zones of Chagas Disease. Sci. Rep..

[4] Keesing F., Holt R.D., Ostfeld R.S. (2006). Effects of Species Diversity on Disease Risk. Ecol. Lett..

[5] Álvarez-Hernández D.-A., Franyuti-Kelly G.-A., Díaz-López-Silva R., González-Chávez A.-M., González-Hermosillo-Cornejo D., Vázquez-López R. (2018). Chagas Disease: Current Perspectives on a Forgotten Disease. Rev. Médica Del. Hosp. Gen. México.

[6] Benjamin R.J., Stramer S.L., Leiby D.A., Dodd R.Y., Fearon M., Castro E. (2012). *Trypanosoma cruzi* Infection in North America and Spain: Evidence in Support of Transfusion Transmission (CME). Transfusion.

[7] Shikanai-Yasuda M.A., Carvalho N.B. (2012). Oral Transmission of Chagas Disease. Clin. Infect. Dis..

[8] Lent H., Wygodzinsky P.W. (1979). Revision of the Triatominae (Hemiptera, Reduviidae), and Their Significance as Vectors of Chagas’ Disease. Bulletin of the AMNH; v. 163, Article 3. Bull. Am. Mus. Nat. Hist..

[9] Galvão C., Carcavallo R., Rocha D.D.S., Jurberg J. (2003). A Checklist of the Current Valid Species of the Subfamily Triatominae Jeannel, 1919 (Hemiptera, Reduviidae) and Their Geographical Distribution, with Nomenclatural and Taxonomic Notes. Zootaxa.

[10] Salazar Schettino P.M., de Haro Arteaga I., Cabrera Bravo M. (2005). Tres Especies de Triatominos y Su Importancia Como Vectores de *Trypanosoma cruzi* En México. Med. Buenos Aires.

[11] Otálora-Luna F., Pérez-Sánchez A.J., Sandoval C., Aldana E. (2015). Evolution of Hematophagous Habit in Triatominae (Heteroptera: Reduviidae). Rev. Chil. Hist. Nat..

[12] Ramsey J.M., Ordoñez R., Cruz-Celis A., Alvear A.L., Chavez V., Lopez R., Pintor J.R., Gama F., Carrillo S. (2000). Distribution of Domestic Triatominae and Stratification of Chagas Disease Transmission in Oaxaca, Mexico. Med. Vet. Entomol..

[13] Martínez-Ibarra J.A., Nogueda-Torres B., García-Benavídez G., Vargas-Llamas V., Bustos-Saldaña R., Montañez-Valdez O.D. (2012). Bionomics of Populations of *Meccus pallidipennis* (Stål), 1872 (Hemiptera: Reduviidae) from Mexico. J. Vector Ecol..

[14] Mesquita R.D., Vionette-Amaral R.J., Lowenberger C., Rivera-Pomar R., Monteiro F.A., Minx P., Spieth J., Carvalho A.B., Panzera F., Lawson D. (2015). Genome of *Rhodnius prolixus*, an Insect Vector of Chagas Disease, Reveals Unique Adaptations to Hematophagy and Parasite Infection. Proc. Natl. Acad. Sci. USA.

[15] Gutiérrez-Cabrera A.E., Córdoba-Aguilar A., Zenteno E., Lowenberger C., Espinoza B. (2016). Origin, Evolution and Function of the Hemipteran Perimicrovillar Membrane with Emphasis on Reduviidae That Transmit Chagas Disease. Bull. Entomol. Res..

[16] Ouali R., Vieira L.R., Salmon D., Bousbata S. (2021). Early Post-Prandial Regulation of Protein Expression in the Midgut of Chagas Disease Vector *Rhodnius prolixus* Highlights New Potential Targets for Vector Control Strategy. Microorganisms.

[17] Kleffmann T., Schmidt J., Schaub G.A. (1998). Attachment of *Trypanosoma cruzi* Epimastigotes to Hydrophobic Substrates and Use of This Property to Separate Stages and Promote Metacyclogenesis. J. Eukaryot. Microbiol..

[18] Garcia E.S., Genta F.A., de Azambuja P., Schaub G.A. (2010). Interactions between Intestinal Compounds of Triatomines and *Trypanosoma cruzi*. Trends Parasitol..

[19] Azambuja P., Garcia E.S., Waniek P.J., Vieira C.S., Figueiredo M.B., Gonzalez M.S., Mello C.B., Castro D.P., Ratcliffe N.A. (2017). *Rhodnius prolixus*: From Physiology by Wigglesworth to Recent Studies of Immune System Modulation by *Trypanosoma cruzi* and *Trypanosoma rangeli*. J. Insect Physiol..

[20] Schaub G.A., Meiser C.K., Balczun C., Mehlhorn H. (2011). Interactions of *Trypanosoma cruzi* and Triatomines. Progress in Parasitology.

[21] Ferreira R.C., Kessler R.L., Lorenzo M.G., Paim R.M.M., Ferreira L.D.L., Probst C.M., Alves-Silva J., Guarneri A.A. (2016). Colonization of *Rhodnius prolixus* Gut by *Trypanosoma cruzi* Involves an Extensive Parasite Killing. Parasitology.

[22] Kollien A., Schaub G. (2000). The Development of *Trypanosoma cruzi* in Triatominae. Parasitol. Today.

[23] Parodi-Talice A., Monteiro-Goes V., Arrambide N., Avila A.R., Duran R., Correa A., Dallagiovanna B., Cayota A., Krieger M., Goldenberg S. (2007). Proteomic Analysis of Metacyclic Trypomastigotes Undergoing *Trypanosoma cruzi* Metacyclogenesis. J. Mass Spectrom..

[24] Perlowagora-Szumlewicz A., Muller C.A. (1982). Studies in Search of a Suitable Experimental Insect Model for Xenodiagnosis of Hosts with Chagas’ Disease. 1—Comparative Xenodiagnosis with Nine Triatomine Species of Animals with Acute Infections by *Trypanosoma cruzi*. Mem. Inst. Oswaldo Cruz.

[25] Schaub G.A., Kleffmann T., Kollien A.H., Schmidt J. (1998). Hydrophobic Attachment of *Trypanosoma cruzi* to the Rectal Cuticle of Triatoma Infestans and Its Influence on Metacyclogenesis—A Review. Tokai J. Exp. Clin. Med..

[26] Engel J.C., Parodi A.J. (1985). *Trypanosoma cruzi* Cells Undergo an Alteration in Protein N-Glycosylation upon Differentiation. J. Biol. Chem..

[27] Azambuja P., Ratcliffe N.A., Garcia E.S. (2005). Towards an Understanding of the Interactions of *Trypanosoma cruzi* and *Trypanosoma rangeli* within the Reduviid Insect Host *Rhodnius prolixus*. An. Acad. Bras. Ciênc..

[28] Chrispeels M.J., Raikhel N.V. (1991). Lectins, Lectin Genes, and Their Role in Plant Defense. Plant Cell.

[29] Li H.-M., Margam V., Muir W.M., Murdock L.L., Pittendrigh B.R. (2007). Changes in *Drosophila melanogaster* Midgut Proteins in Response to Dietary Bowman–Birk Inhibitor. Insect Mol. Biol..

[30] Macedo M.L.R., Freire M.D.G.M., Da Silva M.B.R., Coelho L.C.B.B. (2007). Insecticidal Action of *Bauhinia monandra* Leaf Lectin (BmoLL) against *Anagasta kuehniella* (Lepidoptera: Pyralidae), *Zabrotes subfasciatus* and *Callosobruchus maculatus* (Coleoptera: Bruchidae). Comp. Biochem. Physiol. Part A Mol. Integr. Physiol..

[31] Gutiérrez-Cabrera A.E., Zandberg W.F., Zenteno E., Rodríguez M.H., Espinoza B., Lowenberger C. (2019). Glycosylation on Proteins of the Intestine and Perimicrovillar Membrane of *Triatoma* (*Meccus*) *pallidipennis*, under Different Feeding Conditions. Insect Sci..

[32] Torres-Gutiérrez E., Candelas-Otero F.N., Reynoso-Ducoing O.A., González-Rete B., Vences-Blanco M.O., Cabrera-Bravo M., Bucio-Torres M.I., Salazar-Schettino P.M.S. (2025). Glycosylation Patterns in *Meccus (Triatoma) pallidipennis* Gut: Implications for the Development of Vector Control Strategies. Microorganisms.

[33] Mello C.B., Garcia E.S., Ratcliffe N.A., Azambuja P. (1995). *Trypanosoma cruzi* and *Trypanosoma rangeli*: Interplay with Hemolymph Components of *Rhodnius prolixus*. J. Invertebr. Pathol..

[34] Ratcliffe N.A., Nigam Y., Mello C.B., Garcia E.S., Azambuja P. (1996). *Trypanosoma cruzi* and Erythrocyte Agglutinins: A Comparative Study of Occurrence and Properties in the Gut and Hemolymph of *Rhodnius prolixus*. Exp. Parasitol..

[35] González-Rete B., Gutiérrez-Cabrera A.E., De Fuentes-Vicente J.A., Salazar-Schettino P.M., Cabrera-Bravo M., Córdoba-Aguilar A. (2021). Higher Temperatures Reduce the Number of *Trypanosoma cruzi* Parasites in the Vector *Triatoma pallidipennis*. Parasites Vectors.

[36] Favila-Ruiz G., Jiménez-Cortés J.G., Córdoba-Aguilar A., Salazar-Schettino P.M., Gutiérrez-Cabrera A.E., Pérez-Torres A., De Fuentes-Vicente J.A., Vences-Blanco M.O., Bucio-Torres M.I., Flores-Villegas A.L. (2018). Effects of *Trypanosoma cruzi* on the Phenoloxidase and Prophenoloxidase Activity in the Vector *Meccus pallidipennis* (Hemiptera: Reduviidae). Parasites Vectors.

[37] González-Rete B., Salazar-Schettino P.M., Bucio-Torres M.I., Córdoba-Aguilar A., Cabrera-Bravo M. (2019). Activity of the Prophenoloxidase System and Survival of Triatomines Infected with Different *Trypanosoma cruzi* Strains under Different Temperatures: Understanding Chagas Disease in the Face of Climate Change. Parasit. Vectors.

[38] Mendoza-Rodríguez M. (2015). Caracterización Biológica y Bioquímica de Cuatro Aislados de *Trypanosoma cruzi*. Bachelor’s Thesis.

[39] Zeledón R. (1997). Infection of the Insect Host by *Trypanosoma cruzi*. Atlas of Chagas Disease Vectors in the Americas.

[40] Ambrosio J.R., Reynoso-Ducoing O., Hernández-Sanchez H., Correa-Piña D., González-Malerva L., Cruz-Rivera M., Flisser A. (2003). Actin Expression in *Taenia solium* Cysticerci (Cestoda): Tisular Distribution and Detection of Isoforms. Cell Biol. Int..

[41] Deatherage Kaiser B.L., Wunschel D.S., Sydor M.A., Warner M.G., Wahl K.L., Hutchison J.R. (2015). Improved Proteomic Analysis Following Trichloroacetic Acid Extraction of Bacillus Anthracis Spore Proteins. J. Microbiol. Methods.

[42] Smith P.K., Krohn R.I., Hermanson G.T., Mallia A.K., Gartner F.H., Provenzano M.D., Fujimoto E.K., Goeke N.M., Olson B.J., Klenk D.C. (1985). Measurement of Protein Using Bicinchoninic Acid. Anal. Biochem..

[43] Laemmli U.K. (1970). Cleavage of Structural Proteins during the Assembly of the Head of Bacteriophage T4. Nature.

[44] O’Farrell P. (1975). High Resolution Two-Dimensional Electrophoresis of Proteins. J. Biol. Chem..

[45] Haro I. (1997). de Enfermedad de Chagas En Una Comunidad Del Altiplano Mexicano. Ph.D. Thesis.

[46] Gumiel M., de Mattos D.P., Vieira C.S., Moraes C.S., Moreira C.J.D.C., Gonzalez M.S., Teixeira-Ferreira A., Waghabi M., Azambuja P., Carels N. (2020). Proteome of the Triatomine Digestive Tract: From Catalytic to Immune Pathways; Focusing on Annexin Expression. Front. Mol. Biosci..

[47] Nava-Mirafuentes I. (2015). Proteoma del estómago de *Meccus pallidipennis* (reduviidae, triatominae) asociado a la infección por *Trypanosoma cruzi*. Master's Thesis.

[48] Ribeiro J.M.C., Genta F.A., Sorgine M.H.F., Logullo R., Mesquita R.D., Paiva-Silva G.O., Majerowicz D., Medeiros M., Koerich L., Terra W.R. (2014). An Insight into the Transcriptome of the Digestive Tract of the Bloodsucking Bug, *Rhodnius prolixus*. PLoS Negl. Trop. Dis..

[49] Reynoso-Ducoing O.A., González-Rete B., Díaz E., Candelas-Otero F.N., López-Aviña J.A., Cabrera-Bravo M., Bucio-Torres M.I., Torres-Gutiérrez E., Salazar-Schettino P.M. (2023). Expression of Proteins, Glycoproteins, and Transcripts in the Guts of Fasting, Fed, and *Trypanosoma cruzi*-Infected Triatomines: A Systematic Review. Pathogens.

[50] Nogueira N.P., Saraiva F.M.S., Sultano P.E., Cunha P.R.B.B., Laranja G.A.T., Justo G.A., Sabino K.C.C., Coelho M.G.P., Rossini A., Atella G.C. (2015). Proliferation and Differentiation of *Trypanosoma cruzi* inside Its Vector Have a New Trigger: Redox Status. PLoS ONE.

[51] Garcia E.S., Ratcliffe N.A., Whitten M.M., Gonzalez M.S., Azambuja P. (2007). Exploring the Role of Insect Host Factors in the Dynamics of *Trypanosoma cruzi–Rhodnius prolixus* Interactions. J. Insect Physiol..

[52] Moreira C.J.D.C., De Cicco N.N.T., Galdino T.S., Feder D., Gonzalez M.S., Miguel R.B., Coura J.R., Castro H.C., Azambuja P., Atella G.C. (2018). Lipoproteins from Vertebrate Host Blood Plasma Are Involved in *Trypanosoma cruzi* Epimastigote Agglutination and Participate in Interaction with the Vector Insect, *Rhodnius prolixus*. Exp. Parasitol..

[53] Lander N., Chiurillo M.A., Docampo R. (2021). Signaling Pathways Involved in Environmental Sensing in *Trypanosoma cruzi*. Mol. Microbiol..

[54] Jacobson R.L., Doyle R.J. (1996). Lectin-Parasite Interactions. Parasitol. Today.

[55] Vandenborre G., Smagghe G., Ghesquière B., Menschaert G., Nagender Rao R., Gevaert K., Van Damme E.J.M. (2011). Diversity in Protein Glycosylation among Insect Species. PLoS ONE.

[56] Singh H., Sai P. (2012). Insight of Lectins-A Review. IJSER.

[57] Walski T., De Schutter K., Van Damme E.J.M., Smagghe G. (2017). Diversity and Functions of Protein Glycosylation in Insects. Insect Biochem. Mol. Biol..

[58] Amino R., Serrano A.A., Morita O.M., Pereira-Chioccola V.L., Schenkman S. (1995). A Sialidase Activity in the Midgut of the Insect *Triatoma infestans* Is Responsible for the Low Levels of Sialic Acid in *Trypanosoma cruzi* Growing in the Insect Vector. Glycobiology.

[59] Alves C.R., Albuquerque-Cunha J.M., Mello C.B., Garcia E.S., Nogueira N.F., Bourguingnon S.C., de Souza W., Azambuja P., Gonzalez M.S. (2007). *Trypanosoma cruzi*: Attachment to Perimicrovillar Membrane Glycoproteins of *Rhodnius prolixus*. Exp. Parasitol..

[60] Albuquerque-Cunha J.M., Gonzalez M.S., Garcia E.S., Mello C.B., Azambuja P., Almeida J.C.A., de Souza W., Nogueira N.F.S. (2009). Cytochemical Characterization of Microvillar and Perimicrovillar Membranes in the Posterior Midgut Epithelium of *Rhodnius prolixus*. Arthropod Struct. Dev..

[61] Gutiérrez-Cabrera A.E., Alejandre-Aguilar R., Hernández-Martínez S., Espinoza B. (2014). Development and Glycoprotein Composition of the Perimicrovillar Membrane in *Triatoma (Meccus) pallidipennis* (Hemiptera: Reduviidae). Arthropod Struct. Dev..

[62] Schaub G.A. (2009). Chapter 4 Interactions of Trypanosomatids and Triatomines. Advances in Insect Physiology.

[63] Kwok R., Chung D., Brugge V.T., Orchard I. (2005). The Distribution and Activity of Tachykinin-Related Peptides in the Blood-Feeding Bug, *Rhodnius prolixus*. Peptides.

[64] Sarkar N.R.S., Tobe S.S., Orchard I. (2003). The Distribution and Effects of Dippu-Allatostatin-like Peptides in the Blood-Feeding Bug, *Rhodnius prolixus*. Peptides.

[65] Cámara M.M., Balouz V., Centeno Cameán C., Cori C.R., Kashiwagi G.A., Gil S.A., Macchiaverna N.P., Cardinal M.V., Guaimas F., Lobo M.M. (2019). *Trypanosoma cruzi* Surface Mucins Are Involved in the Attachment to the Triatoma Infestans Rectal Ampoule. PLoS Negl. Trop. Dis..

[66] Garcia E.S., Azambuja P. (1991). Development and Interactions of *Trypanosoma cruzi* within the Insect Vector. Parasitol. Today.

[67] Palau Castaño M.T. (1996). Estudio experimental del impacto de la estructura clonal de “*Trypanosoma cruzi*” sobre aspectos médico-biológicos. Ph.D. Thesis.

[68] Melo R.D.F.P., Guarneri A.A., Silber A.M. (2020). The Influence of Environmental Cues on the Development of *Trypanosoma cruzi* in Triatominae Vector. Front. Cell. Infect. Microbiol..

[69] Salazar-Schettino M.P., Rojas-Wastavino G.E., Cabrera-Bravo M., Bucio-Torres M.I., Martínez-Ibarra J.A., Monroy-Escobar M.C., Rodas-Retana A., Guevara-Gómez Y., Vences-Blanco M.O., Ruiz Hernández A.L. (2010). Revisión de 13 especies de la familia *Triatominae* (Hemiptera: Reduviidae) vectores de la enfermedad de Chagas, en México. J. Selva Andin. Res. Soc..

[70] Cortés-Jiménez M., Nogueda-Torres B., Alejandre-Aguilar R., Isita-Tornell L., Ramírez-Moreno E. (1996). Frequency of Triatomines Infected with *Trypanosoma cruzi* Collected in Cuernavaca City, Morelos, México. Rev. Latinoam. Microbiol..

[71] Martínez Ibarra J.A., Novelo López M. (2004). Blood meals to molt, feeding time and postfeeding defecation delay of *Meccus pallidipennis* (Stål, 1872) (Hemiptera: Reduviidae) under laboratory conditions. Folia Entomológica Mex..

[72] Martínez-Ibarra J.A., Alejandre-Aguilar R., Torres-Morales A., Trujillo-García J.C., Nogueda-Torres B., Trujillo-Contreras F. (2006). Biology of Three Species of the *Meccus phyllosomus* Complex (Hemiptera: Reduviidae: Triatominae) Fed on Blood of Hens and Rabbits. Mem. Inst. Oswaldo Cruz.

[73] Franzim-Junior E., Mendes M.T., Anhê A., da Costa T.A., Silva M., Hernandez C., Pelli A., Sales-Campos H., Oliveira C. (2018). Biology of *Meccus pallidipennis* (Hemiptera: Reduviidae) to Other Conditions than That Encountered in Their Native Habitat. J. Arthropod Borne Dis..

[74] Guarneri A.A., Carvalho M.D.G., Pereira M.H., Diotaiuti L. (2000). Potencial Biológico Do *Triatoma brasiliensis*. Cad. Saúde Pública.

[75] Christensen H.A., Sousa O.E., De Vasquez A.M. (1988). Host Feeding Profiles of *Triatoma dimidiata* in Peridomestic Habitats of Western Panama. Am. J. Trop. Med. Hyg..

[76] Martínez-Ibarra J.A., Nogueda-Torres B., Salazar-Schettino P.M., Vences-Blanco M.O., de la Torre-Álvarez F.J., Montañez-Valdez O.D. (2014). Differences on Biological Attributes of Three Populations of *Meccus pallidipennis* Stål (Hemiptera: Reduviidae). J. Vector Borne Dis..

